# Effects of urbanization and lifestyle habits on the intestinal microbiota of adolescents in eastern China

**DOI:** 10.3389/fmicb.2023.989303

**Published:** 2023-06-12

**Authors:** Gang Zhao, Lu Xie, Yan Wu, Bing Wang, Weilin Teng, Zhou Sun, Qingjun Kao, Wei Liu, Xionge Pi, Haiyan Ma

**Affiliations:** ^1^Department of Infectious Disease Control and Prevention, Hangzhou Center for Disease Control and Prevention, Hangzhou, China; ^2^School of Public Health, Hangzhou Normal University, Hangzhou, China; ^3^Zhejiang Academy of Agriculture Sciences, Institute of Plant Protection and Microbiology, Hangzhou, China

**Keywords:** adolescents, urbanization, intentional microbiota, lifestyle habits, China

## Abstract

**Introduction:**

Owing to urbanization, living habits have changed widely, leading to alterations in the intestinal microbiota of urban residents. However, there are few studies on the characteristics of intestinal microbiota of adolescents living in different urbanized areas in China.

**Methods:**

A total of 302 fecal samples collected from adolescent students in eastern China were examined. 16S rRNA high-throughput sequencing was used to identify the fecal microbiota. These data were combined with questionnaire survey results to investigate the effect of urbanization on the intestinal microbiota of adolescents in eastern China. Moreover, the role of lifestyle habits in this relationship was also evaluated.

**Results:**

The results revealed significant differences in the structure of the intestinal microbiota among adolescents living in regions with different levels of urbanization. Adolescents living in urban regions had a significantly higher proportion of *Bacteroides* (*p* <  0.001, FDR = 0.004), whereas those living in towns and rural regions had higher proportions of *Bifidobacterium* (*p* < 0.001, FDR < 0.001) and *Prevotella* (*p*  < 0.05, FDR = 0.019). The diversity of the intestinal microbiota was higher in urban residents than in adolescents living in towns and rural regions (*p*  <  0.05). In addition, the differences in intestinal microbiota across individuals living in cities, towns, and rural regions were related to dietary preferences, flavor preferences, and sleep and exercise durations. Adolescents who ate more meat had more *Dorea* (LDA = 3.622, *p* = 0.04), while the abundance of *Escherichia*–*Shigella* is higher among adolescents who ate more condiments (LDA = 4.285, *p* = 0.02). The abundance of *Dialister* was significantly increased in adolescents with longer sleep durations (LDA = 4.066, *p =* 0.03). Adolescents who exercised for a long duration had more *Faecalibacterium* than those who exercised for a shorter duration (LDA = 4.303, *p* = 0.04).

**Discussion:**

Our research has preliminarily demonstrated that there were differences in the composition of Gut microbiome in stool samples of adolescents living in different urbanized areas, and provide a scientific basis for the maintenance of a healthy intentional microbota in adolescences.

## 1. Introduction

The human gastrointestinal tract contains 200–300 square meters of mucous membrane, which hosts more than 1,000 types of microorganisms with a total weight of 1–1.5 kg. These microorganisms, dispersed across 50 bacterial phyla, constitute the human intestinal microbiota (Qin et al., 2010; Backhed et al., 2015).

The intestinal microbiota plays an important role in health. Studies have shown that the intestinal microbiota influences the body’s metabolism, immune system, and brain function (Turnbaugh et al., 2007; Cho and Blaser, 2012; Shreiner et al., 2015). The gastrointestinal microbiota promotes the digestion of dietary fibers and improves lipid metabolism by inhibiting lipase activity in adipocytes (Hooper et al., 2001). In addition, the intestinal mucosa plays an important role in immunity, protecting the intestinal epithelium from the direct attachment of microorganisms. The intestinal microbiota can promote the maturation of the mucosal layer and improve immunity *via* FoxP3 + CD4 + cells (Nagano et al., 2012). There also exists a bidirectional network of communication between the intestinal microbiota and the brain called the gut–brain axis. Short-chain fatty acids, which are bacterial metabolites, affect the integrity of the blood–brain barrier by increasing the production of tight junction proteins. This, in turn, activates neurons and alters neurological function, leading to emotional and behavioral changes (Gomaa, 2020; Morais et al., 2021).

Previous studies have demonstrated a strong connection between the intestinal microbiota and gut disorders. Irritable bowel syndrome (IBS) and inflammatory bowel disease (IBD) are known to be caused by imbalances in the intestinal microbiota, leading to the growth of pathogenic microorganisms and subsequent damage to the intestinal mucosa (Ghoshal et al., 2012; Parekh et al., 2015). In addition, the intestinal microbiota plays an important role in host energy metabolism. Previous studies have revealed significant differences in the intestinal microbiota between healthy individuals and patients with obesity, fatty liver disease, diabetes, cardiovascular disease, and other diseases (Gomaa, 2020). Obese individuals show a higher proportion of *Porphyromonas*, *Campylobacter*, *Bacteroides*, *Staphylococcus*, and *Parabacteroides* and a decrease in the proportion of probiotic bacteria (Le Chatelier et al., 2013; Crovesy et al., 2020).

The intestinal microbiota is also related to immune and nervous system diseases. The intestinal microbiota of patients with allergy and asthma is significantly different from that of healthy individuals (Frati et al., 2018). Dysfunction of the brain–gut axis is associated with many neurological and psychiatric disorders, including stress-related disorders such as anxiety and depression, neurodevelopmental disorders such as autism, and neurodegenerative diseases such as Alzheimer’s disease (Clarke et al., 2013; Kelly et al., 2016; Sherwin et al., 2016; Strati et al., 2017). Studies have also shown that probiotic regimens can have a significant therapeutic effect on these psychiatric disorders (McKean et al., 2017). Taken together, this evidence suggests that more attention should be paid to gut health and the maintenance of a healthy intestinal microbiota, which is important for overall health.

In humans, the intestinal microbiota changes with age. Previously, it was believed that the structure of the intestinal microbiota in 3-year-old children was similar to that in adults (Chana Palmer et al., 2007). However, accumulating evidence shows that the development of the human intestinal microbiota is not as rapid as previously described. Instead, the intestinal microbiota gradually matures with age and even during adolescence (Kim et al., 2018; Vijayakumar et al., 2018). Adolescence is a critical period of growth and development. Physical development during adolescence lays the foundation for health in adulthood. Adolescents experience rapid physical changes, including changes in their body shape, sexual development and maturation, brain and nerve development, and increased energy metabolism (Golden et al., 2014; Vijayakumar et al., 2018). Thus, maintaining a healthy intestinal microbiota could promote the absorption of nutrients, prevent diseases, and improve physical development in adolescents, laying the foundation for good overall health in adulthood.

Urbanization is closely related to a decrease in the diversity of the human microbiome. Thus, the intestinal microbiota of adolescents living in regions with different levels of urbanization may also differ, affecting their growth and development at this stage. In previous studies, it was found that *Bacteroides* was more abundant in the intestines of adolescents living in cities, while *Prevotella* was more abundant in adolescents living in rural areas (Nakayama et al., 2015). *Bacteroides* can maintain intestinal stability by producing short-chain fatty acids (Tian et al., 2017). The decrease of *Prevotella* has been proved to be related to many diseases (Moon et al., 2017). Maintaining the balance of intestinal microecology is of great significance to health. Therefore, analyzing the differences in the intestinal microbiota in adolescents from areas with different levels of urbanization is of great significance for helping them maintain a healthy intestinal microenvironment.

Further, economic gaps contribute to variations in the environments and living habits of urban residents, thus influencing their intestinal microbiota. According to previous studies, differences in economic status and dietary habits have resulted in large differences in the intestinal microbiota of individuals from the East and West (Yatsunenko et al., 2012). The fecal samples of westerners show a high proportion of *Bacteroides* owing to the consumption of more high-fat meat. However, individuals in the East show a higher proportion of *Prevotella* because they consume more high-fiber carbohydrates (Nakayama et al., 2015). In addition, behavioral habits such as exercise and sleep durations can also affect the intestinal microbiota. Studies have reported that exercise has a significant impact on the intestinal microbiota in adolescents. Further, after exercise interventions, the intestinal microbiota of children with diabetes appears similar to that of healthy children (Quiroga et al., 2020). In breast cancer survivors, sleep disturbances are linked to the abundance of *Paracoccus*, *Rikenellaceae*, and *Clostridium* groups (Paulsen et al., 2017). Moreover, evidence suggests that metabolic disorders associated with sleep deprivation could be mediated by the overgrowth of specific intestinal bacteria (Matenchuk et al., 2020).

However, few previous studies have found links between living habits and urbanization or explored the reasons for variations in the intestinal microbiota among residents of areas with different urbanization levels.

This study investigated the characteristics of the intestinal microbiota in adolescents from regions in Hangzhou with different levels of urbanization. We analyzed the effects of diet, exercise and sleep on these characteristics to provide a scientific basis for the maintenance of intestinal health in adolescents from regions with different levels of urbanization.

## 2. Materials and methods

### 2.1. Study population and collection of fecal samples

Using stratified cluster sampling, five districts/counties in Hangzhou were selected: Shangcheng District, Gongshu District, Xihu District, Fuyang District, and Chun‘an County. Four schools—including two junior high schools and two primary schools—were selected from each district. Subsequently, one class with more than 16 students was selected from each school.

According to the urbanization level of different districts/counties, the regions were divided into three groups: urban, town, and rural areas. The urban group was composed of students from Shangcheng District and Gongshu District; the town group was composed of students from four schools in Xihu District and the adolescents from the town in Fuyang District; and the rural group was composed of the students from Chun’an County and the adolescents from the rural region in Fuyang District. Adolescents aged 7–15 years (contained the younger age) were included in this study and referred to the national health industry standard WS/T 610-2018. Exclusion criteria: Antibiotics have been used in the past 15 days (Qin et al., 2010; Backhed et al., 2015). Gastrointestinal dysfunction, previous gastrointestinal disease history (Shreiner et al., 2015). Diarrhea, abdominal distension, abdominal pain or constipation within the past 15 days. Finally, 302 adolescents were included in the study.

Fecal samples were obtained from the participants and stored at 4°C immediately following collection and sent to the laboratory within 4 h and frozen for DNA sample extraction. This research was approved by the Ethics Committee of Hangzhou Center for Disease Control and Prevention (No. 20047), and written informed consent was obtained from the parents or guardians of each adolescent.

### 2.2. Questionnaire design

The questionnaire contained questions on basic demographic information such as sex, age, and grade. Lifestyle habits of nearly 7 days, such as dietary preferences, flavor preferences, sleep durations, and exercise durations were also surveyed. The questionnaire was self-evaluated. In order to prevent adolescents from providing inaccurate responses while filling out their questionnaire owing to a lack of question comprehension or recall bias, they were accompanied by their parents while filling out the questionnaire. Thus, parents and adolescents completed the questionnaire together. Dietary preferences were divided into three categories: 1. Preference for vegetables (Vegetable intake is higher than meat), 2. Preference for meat (Meat intake is higher than vegetables), and 3. Balanced proportion of vegetables and meat. Flavor preferences were divided into three categories: 1. Light flavor, 2. Heavy seasoning (spicy, salty, sweet, sour, etc.), and 3. Balanced flavor. Sleep duration was divided into 1. Less than 7 h, 2. 7–9 h, and 3. More than 9 h. Exercise duration was divided into four groups: 1. Less than 0.5 h, 2. 0.5–1 h, 3. 1–2 h, and 4. More than 2 h.

This questionnaire was designed by project team members based to the opinions of experts, strictly following the principles of probability and statistics. This questionnaire was designed according to “Zhejiang Adult Behavior Risk Factors Monitoring Questionnaire.” It has been pre-investigated, expertly demonstrated, and conducted face-to-face investigations by trained and qualified investigators.

### 2.3. DNA extraction and PCR amplification

Total microbial genomic DNA was extracted from fecal samples using the E.Z.N.A.^®^ soil DNA Kit according to manufacturer’s instructions. The quality and concentration of DNA were determined by 1.0% agarose gel electrophoresis and a NanoDrop^®^ ND-2000 spectrophotometer (Thermo Scientific Inc., United States) and kept at − 80°C prior to further use. The hypervariable region V3–V4 of the bacterial 16S rRNA gene were amplified with primer pairs 806R(5′-GGACTACHVGGGTWTCTAAT-3′; Liu et al., 2016) by an ABI GeneAmp^®^ 9,700 PCR thermocycler (ABI, CA, United States). The PCR reaction mixture including 4 μL 5 × Fast Pfu buffer, 2 μL 2.5 mM dNTPs, 0.8 μL each primer (5 μM), 0.4 μL Fast Pfu polymerase, 10 ng of template DNA, and ddH2O to a final volume of 20 μL. PCR amplification cycling conditions were as follows: initial denaturation at 95°C for 3 min, followed by 27 cycles of denaturing at 95°C for 30 s, annealing at 55°C for 30 s and extension at 72°C for 45 s, and single extension at 72°C for 10 min, and end at 4°C. All samples were amplified in triplicate. The PCR product was extracted from 2% agarose gel and purified using the AxyPrep DNA Gel Extraction Kit (Axygen Biosciences, Union City, CA, United States) according to manufacturer’s instructions and quantified using Quantus^™^ Fluorometer (Promega, United States).

### 2.4. Illumina MiSeq sequencing

Purified amplicons were pooled in equimolar amounts and paired-end sequenced on an Illumina NovaSeq PE250 platform (Illumina, San Diego, United States) according to the standard protocols by Majorbio Bio-Pharm Technology Co. Ltd. (Shanghai, China). The raw sequencing reads were deposited into the NCBI Sequence Read Archive (SRA) database (Accession Number: PRJNA856408).

### 2.5. Amplicon sequence processing and analysis

After demultiplexing, the resulting sequences were quality filtered with fastp (0.19.6; Chen et al., 2018) and merged with FLASH (v1.2.11; Magoč and Salzberg, 2011). Then the high-quality sequences were denoised using DADA2 (Callahan et al., 2016) plugin in the Qiime2 (Bolyen et al., 2019; version 2020.2) pipeline with recommended parameters, which obtains single nucleotide resolution based on error profiles within samples. DADA2 denoised sequences are usually called amplicon sequence variants (ASVs). To minimize the effects of sequencing depth on alpha and beta diversity measure, the number of sequences from each sample was rarefied to 24,635, which still yielded an average Good’ s coverage of 99.10%. Chao1 and Shannon index were calculated with Mothur (v1.30.1; Schloss et al., 2009). Taxonomic assignment of ASVs was performed using the Naive bayes consensus taxonomy classifier implemented in Qiime2 and the SILVA 16S rRNA database (v138).

### 2.6. Statistical analysis

The differences of Alpha diversity index (Chao index and Shannon index) between groups were determined by Kruskal–Wallis rank-sum test. Beta diversity principal coordinate analysis (PCoA) is determined based on ASV table and Bray–Curtis distance algorithm. The abundance of each microbiota at the level of phylum and genus was counted and displayed as Veen diagram and Bar diagram. The comparative analysis of genus intestinal microflora was determined by Kruskal-Wallis rank sum test and Tukey–Kramer back testing, False discovery rates (FDRs) were calculated to account for multiple testing, FDR < 0.05 was considered statistically significant. LEfSe (Linear discriminant analysis Effect Size) online software^1^ was used for linear discriminant analysis (LDA ≥ 2) to determine the bacteria with significant differences in different groups (Segata et al., 2011). Canonical correlation analysis (CCA) combines correspondence analysis with multiple regression analysis, and each step is calculated by regression with environmental factors, usually used to determine the correlation between sample distribution, intestinal microbiota and environmental factors (Mccune, 1997). The above data analysis and mapping are all carried out on the online platform of Majorbio Cloud Platform^2^. IBM SPSS version 21 software was used for statistical analysis, and Chi-square analysis was used to compare demographic characteristics among adolescents. Bonferroni adjustment was used for *post-hoc* testing. Significant difference was defined as *p* < 0.05.

## 3. Results

### 3.1. Basic characteristics of the study population

In total, 302 adolescents were included in the study: 102 from cities areas (age, 11.11 ± 2.746 years), 97 from towns (age, 10.61 ± 2.760 years), and 103 from rural areas (age, 10.21 ± 2.959 years). There were no significant differences in age, grade, and gender among the three regions (Table 1).

**Table 1:** Characteristics of the adolescent population in different regions.

		City	Town	Rural area	*p*
Age (years) (*x* ± *s*)		11.11 ± 2.746	10.61 ± 2.760	10.21 ± 2.959	0.078
Gender	Male	52(51%)	47(48.5%)	55(53.4%)	0.746
	Female	50(49%)	50(51.5%)	48(46.6%)	
Grade	Primary school	49(48%)	52(53.6%)	55(53.4%)	0.668
	Junior school	53(52%)	45(46.4%)	48(46.6%)	
Dietary preference	Vegetables	6(5.9%) b	13(13.4%) a, b	17(16.5%) a	0.048
	Meat	23(22.5%)	31(32.0%)	24(23.3%)	
	Balanced	73(71.6%) a	53(54.6%) b	62(60.2%) a, b	
Flavor preference	Light	12(11.8%) a	9(9.3%) b	24(23.3%) a, b	0.04
	Heavy	22(21.6%)	27(27.8%)	24(23.3%)	
	Balanced	68(66.7%)	61(62.9%)	55(53.4%)	
Sleep duration	<7 h	7(6.9%)	8(8.2%)	2(1.9%)	0.008
	7–9 h	68(66.7%) a	80(82.5%) b	81(78.6%) a, b	
	> 9 h	27(26.5%) a	9(9.3%) b	20(19.4%) a, b	
Exercise duration	< 0.5 h	16(15.7%) b	15(15.5%) b	33(32.0%) a	0.001
	0.5–1 h	52(51.0%) a, b	61(62.9%) b	36(35.0%) a	
	1–2 h	27(26.5%) a	12(12.4%) b	22(21.4%) a, b	
	> 2 h	7(6.9%)	9(9.3%)	12(11.7%)	

^*^a and b: There are significant differences between the two groups; a, b: There is no significant difference between this group and the other two groups.

The dietary and flavor preferences and exercise and sleep durations of adolescents from different regions are shown in Table 1. There were significant differences in these lifestyle habits among the three regions. The proportion of adolescents with a balanced dietary intake was highest in cities, and that of those who consumed more vegetables was the highest in rural areas. Regarding flavor preferences, the proportion of adolescents with a balanced flavor preference was the highest in cities, and adolescents with light flavor in cities are significantly more than those in town. Cities also had the highest proportion of adolescents who slept for more than 9 h. However, adolescents who exercised for more than 2 h a day were the most common in rural areas. These differences in lifestyle habits may have created differences in the intestinal microbiota among adolescents living in different areas of Hangzhou.

### 3.2. Characteristics of the intestinal microbiota based on the level of urbanization

**Figure 1:**
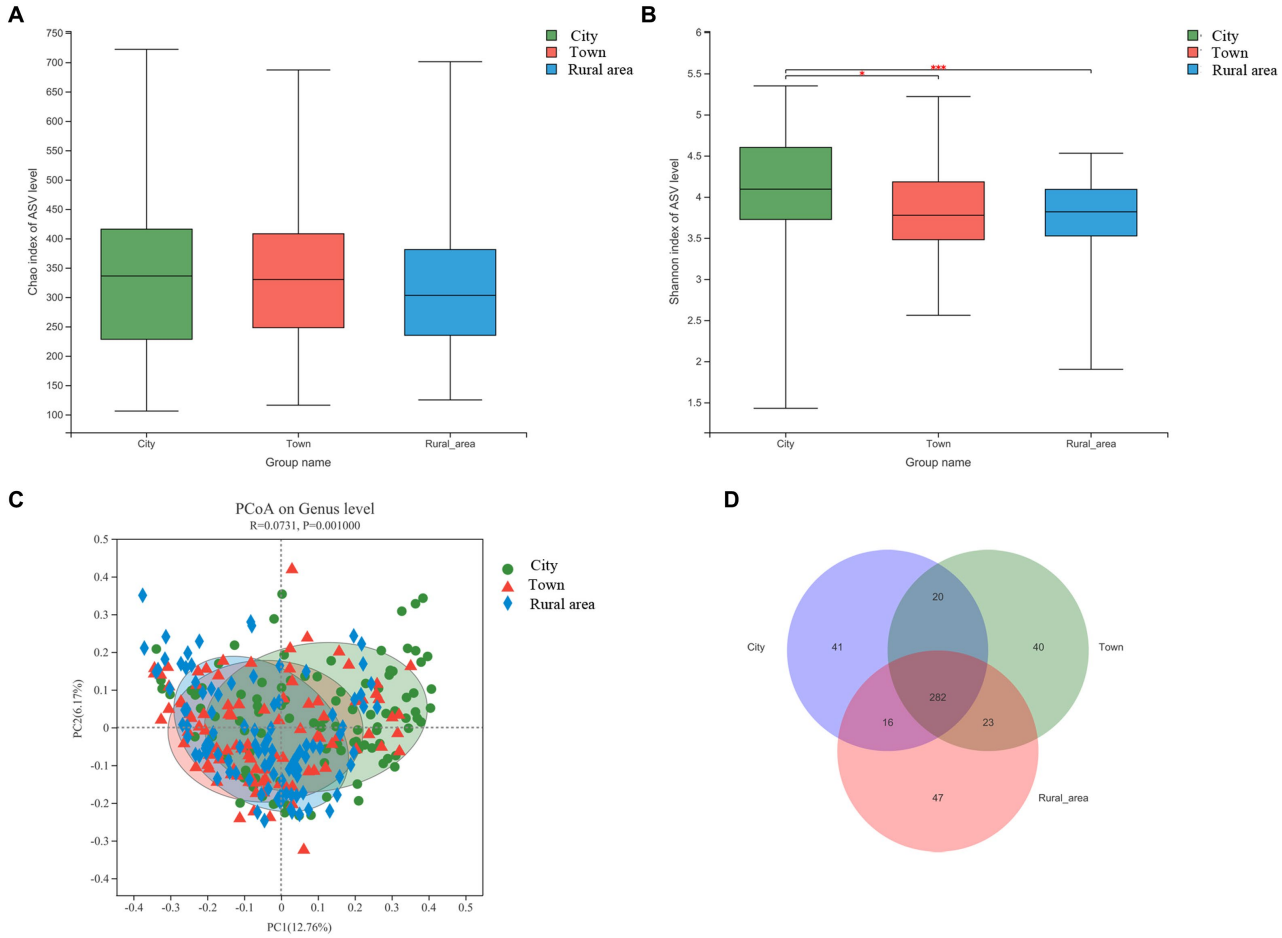
Alpha and beta diversity of the intestinal microbiota from adolescents in different regions, **(A)** Chao index; **(B)** Shannon index; **(C)** Beta diversity; **(D)** Venn diagram. Statistical significance thresholds were: ^*^0.01 < *p* ≤ 0.05; ^**^0.001 < *p* ≤ 0.01; ^***^p ≤ 0.001.

16S rRNA sequencing technology was used to analyze the fecal microbiota in adolescents from different regions. First, the Alpha diversity of the intestinal microbiota in adolescents was calculated using the Wilcoxon rank-sum test. The Chao index reflects the richness of communities in the samples, and the Shannon index reflects their diversity. Our results showed no significant difference in the Chao index among adolescents from different regions. However, the Shannon index was significantly different (*p* < 0.001). The Shannon index was the highest in adolescents from cities, followed by those from towns and rural areas. Hence, the intestinal microbiota of city adolescents was more diverse than that of adolescents from other regions (Figures 1A,B).

Next, we examined the relationship between microbial communities in different regions using PCoA based on Bray-Curtis distances. The results showed that the composition of the intestinal microbiota was significantly different among adolescents from different regions (PERMANOVA, *p* = 0.001; Figure 1C), except between adolescents from town and rural area (*p* = 0.37; Supplementary Figure S1). As shown in the Venn diagram, there were 282 bacterial species shared among residents of cities, towns, and rural areas. However, 41, 40, and 47 bacterial species were exclusive to those from cities, towns, and rural areas, respectively (Figure 1D). Combined with the results of Alpha diversity, the results showed that adolescents from regions of higher urbanization had a more diverse microbiota.

**Figure 2:**
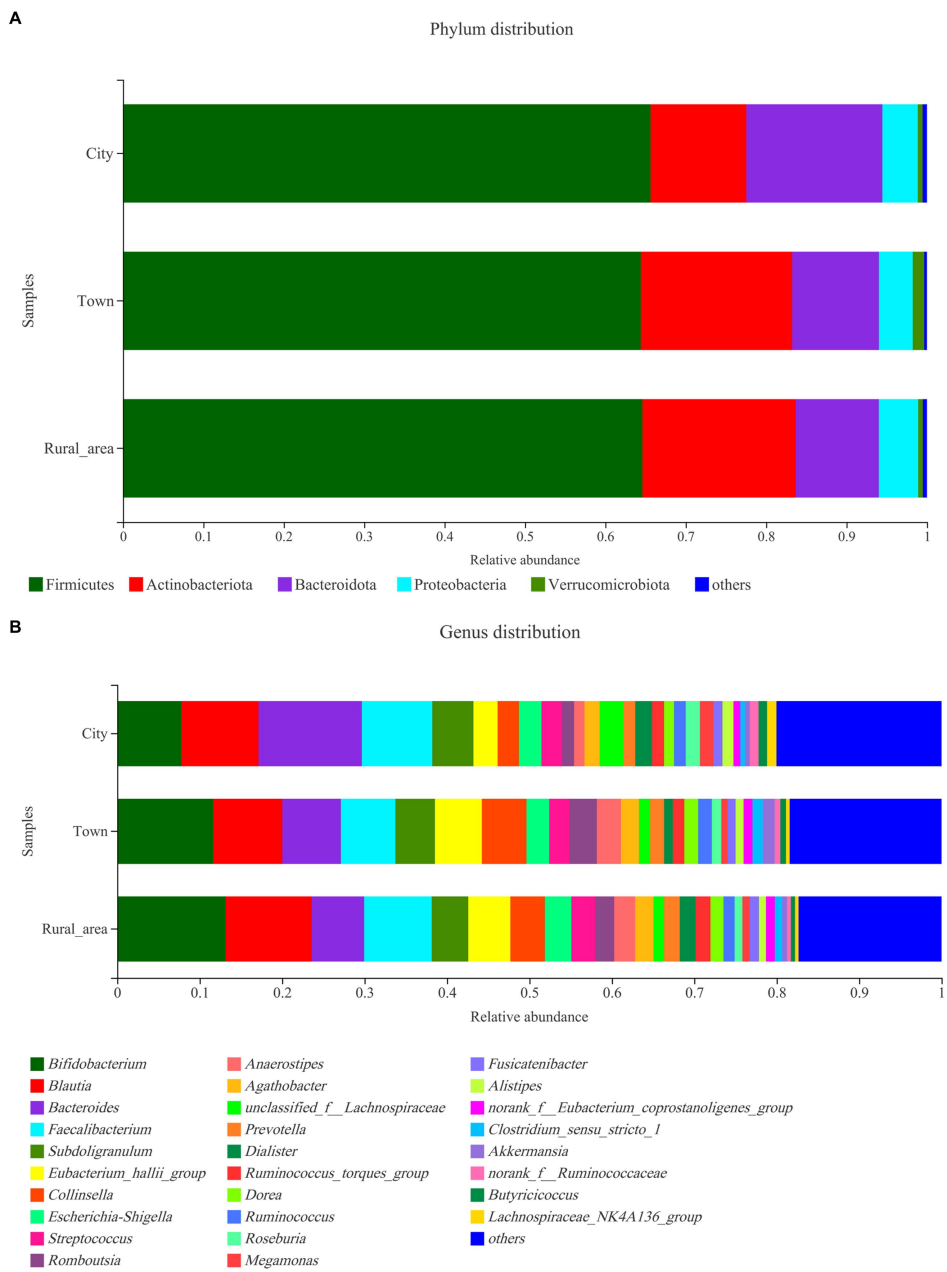
Community distribution of the intestinal microbiota in adolescents from different regions at the phylum and genus levels. **(A)** phylum level; **(B)** genus level.

The distribution of the intestinal microbiota in adolescents from different regions was analyzed at the phylum and genus level using a community bar diagram. The relative abundances of *Firmicutes*, *Actinobacteria*, *Bacteroidota*, and *Proteobacteria* were different among the different regions. In cities, *Firmicutes* accounted for 65.63% of the standardized readings, followed by *Bacteroidota* (16.92%), *Actinobacteria* (11.90%), and *Proteobacteria* (4.41%). In towns, *Firmicutes* accounted for 64.42% of standardized readings, followed by *Actinobacteria* (18.82%), *Bacteroidota* (10.79%), and *Proteobacteria* (4.2%). In rural areas, *Firmicutes* accounted for 64.61% of standardized readings, followed by *Actinobacteria* (19.06%), *Bacteroidota* (10.35%), and *Proteobacteria* (4.89%). Compared with towns and rural areas, the relative abundance of *Bacteroidota* in cities was higher (16.92 vs. 10.79 vs. 10.35%, *p* = 0.003, FDR = 0.027), while the proportion of *Actinobacteria* was lower (11.90 vs. 18.82 vs. 19.06%, *p* < 0.001, FDR < 0.001; Figure 2). The ratios of *Firmicutes* to *Bacteroidota* in cities, towns, and rural areas were 3.88, 5.97, and 6.25, respectively.

**Figure 3:**
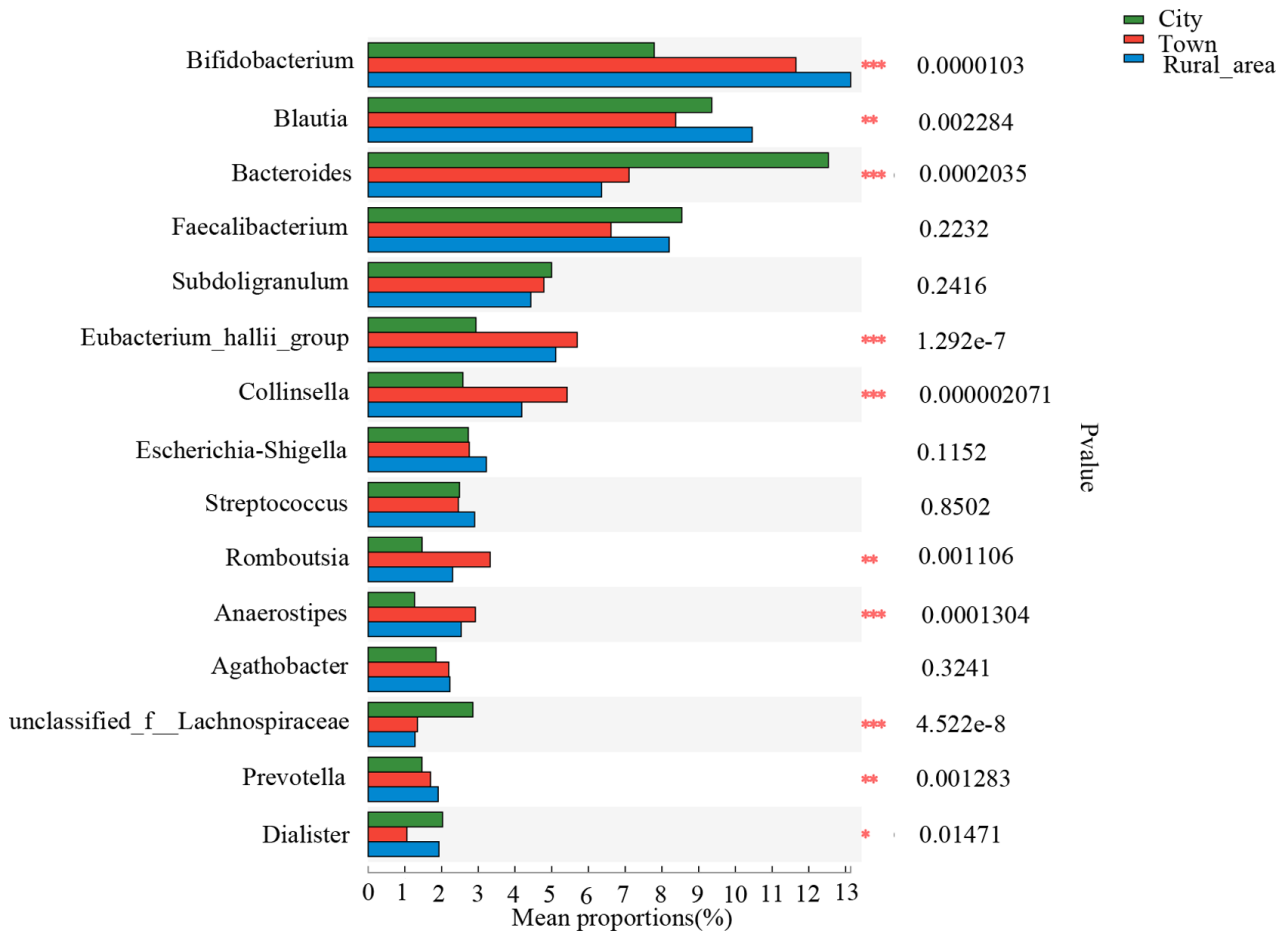
Comparison of the fecal microbiota across different regions at the genus level. Statistical significance thresholds were: ^*^0.01 < *p* ≤ 0.05; ^**^0.001 < *p* ≤ 0.01; ^***^p ≤ 0.001.

The Wilcoxon rank-sum test was used to assess differences in the distribution of gut microbes at the genus level among adolescents from different regions. Among the 15 bacterial genera with the highest relative abundance, 10 genera showed statistical differences in the gut of adolescents from different regions (Figure 3). *Bifidobacterium* was more abundant in the intestinal microbiota of adolescents from rural areas, and the least abundant in those from cities (*p* < 0.001). *Bacteroides* was most commonly found in cities and less commonly in towns and rural areas (*p* < 0.001). *Blautia* and the *Eubacterium hallii* group were more frequent in towns and rural areas than in cities (*p* < 0.001). The abundance of *Collinsella*, *Romboutsia*, *Anaerostipes*, *unclassified_f_Lachnospiraceae*, *Prevotella*, *Dialister* are also significantly different among adolescents in different areas. However, after FDR correction, the statistical significance of *Dialister* disappeared. There was no significant difference in *Escherichia*–*Shigella* among the three regions (Table 2).

**Table 2:** Comparison of the fecal microbiota across different regions at the genus level.

Genus	City	Town	Rural area	*p*	*Q*
*Bifidobacterium*	7.79 ± 10.41	11.65 ± 10.12	13.14 ± 13.5	<0.001	<0.001
*Blautia*	9.356 ± 10.28	8.376 ± 5.031	10.46 ± 7.82	0.002	0.031
*Bacteroides*	12.53 ± 13.86	7.104 ± 8.816	6.357 ± 8.174	<0.001	0.004
*Eubacterium hallii group*	2.936 ± 3.068	5.695 ± 4.967	5.111 ± 4.776	<0.001	<0.001
*Collinsella*	2.58 ± 6.403	5.416 ± 7.41	4.185 ± 5.775	<0.001	<0.001
*Romboutsia*	1.47 ± 2.246	3.323 ± 4.7	2.3 ± 2.295	0.001	0.018
*Anaerostipes*	1.264 ± 1.305	2.92 ± 3.973	2.534 ± 2.701	<0.001	0.003
*unclassified_f__Lachnospiraceae*	2.852 ± 4.719	1.345 ± 1.521	1.279 ± 1.182	<0.001	<0.001
*Prevotella*	1.465 ± 7.561	1.7 ± 5.327	1.91 ± 5.112	0.001	0.019
*Dialister*	1.948 ± 4.369	1.097 ± 2.142	1.927 ± 4.215	0.01	0.128

**Figure 4:**
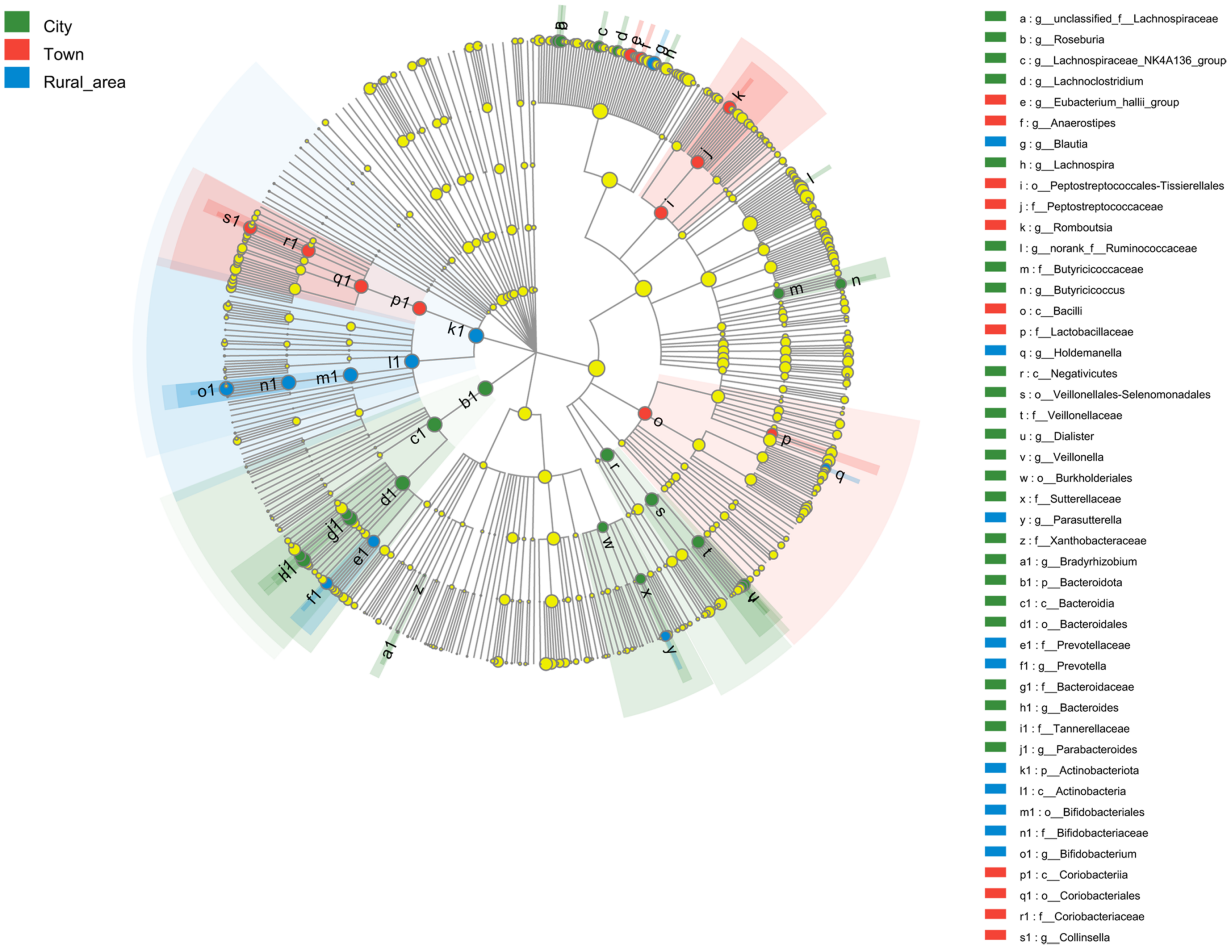
LefSe analysis of the intestinal microbiota from adolescents in different regions.

LEfSe was used to analyze the key intestinal bacteria contributing to community structure differences among adolescents from different regions. The results showed that the intestinal microbiota of adolescents in cities was rich in *Bacteroidales* (LDA = 4.480, *p* = 0.001) order, *Bacteroidia* (LDA = 4.480, *p* = 0.001) class, *Bacteroidota* (LDA = 4.480, *p* = 0.001) phylum, *Bacteroides* (LDA = 4.475, *p* < 0.001) genus, and *Bacteroidaceae* (LDA = 4.475, *p* < 0.001) family. In towns, the abundance of *Coriobacteriales* (LDA = 4.198, *p* < 0.001) order, *Coriobacteriia* (LDA = 4.198, *p* < 0.001) class, *Collinsella* (LDA = 4.157, *p* < 0.001) genus, *Coriobacteriaceae* (LDA = 4.156, *p* < 0.001) family and the *Eubacterium hallii* group (LDA = 4.109, *p* < 0.001) genus was significantly higher than that in other areas. Adolescents from rural areas showed a higher enrichment of *Actinobacteriota* (LDA = 4.548, *p* < 0.001) phylum, *Actinobacteria* (LDA = 4.407, *p* < 0.001) class, *Bifidobacterium* (LDA = 4.405, *p* < 0.001) genus, *Bifidobacteriaceae* (LDA = 4.404, *p* < 0.001) family, *Bifidobacteriales* (LDA = 4.404, *p* < 0.001) order, *Blautia* (LDA = 3.936, *p* = 0.001) genus, *Prevotellaceae* (LDA = 3.828, *p* = 0.001) family, *Prevotella* (LDA = 3.782, *p* = 0.001) genus (Figure 4).

### 3.3. Factors influencing the intestinal microbiota in adolescents from regions with different levels of urbanization

**Figure 5:**
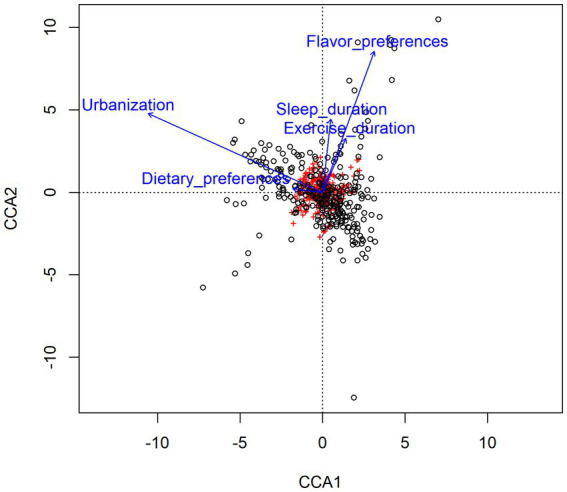
Canonical correlation analyses (CCA) of the correlation between gut microbiota and related environmental factors. Arrows represent different environmental factors, red labels represent genera of gut microbiota, and black circles represent samples. The length of the arrow represents the degree of correlation between the environmental factor and gut microbiota, and the longer the arrow represents the greater the influence on the composition of gut microbiota by related environmental factor. The Angle between the arrow lines represents the direction of the correlation, the acute angle indicates the positive correlation between the two environmental factors, and the obtuse angle is the negative correlation.

**Figure 6:**
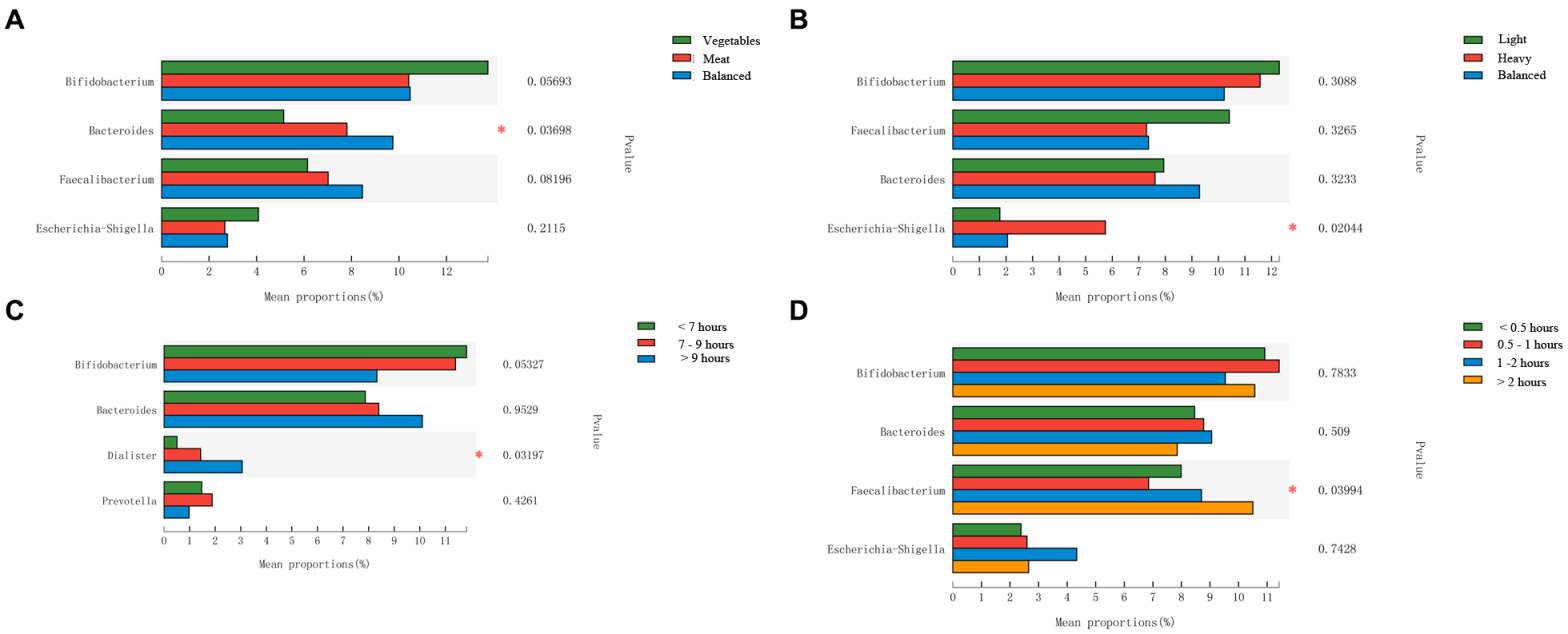
Comparison of the intestinal microbiota from adolescents with different lifestyle habits at the genus level. **(A)** Dietary preference; **(B)** Flavor preference; **(C)** Sleep duration; **(D)**: Exercise Duration. Statistical significance thresholds were: ^*^0.01 < *p* ≤ 0.05; ^**^0.001 < *p* ≤ 0.01; ^***^p ≤ 0.001.

In order to further analyze the impact of lifestyle habits on the relationship between urbanization and intestinal microbiota, CCA (canonical correlation analyses) was conducted between intestinal microbiota and environmental factors. As shown in Figure 5, urbanization and flavor preference are the two factors that have the greatest influence on the intestinal microbiota of adolescents in Hangzhou, and urbanization has a positive correlation with dietary preference and sleep duration, indicating that the differences of intestinal microbiota of adolescents in different areas may be related to their lifestyle habits. The distribution of intestinal microbiota in adolescents with different lifestyle habits was observed based on beta diversity. There were significant differences in the genus-level bacterial community structure (beta diversity) among adolescents with different sleep durations (Supplementary Figure S2). We used the Wilcoxon rank-sum test to analyze the characteristics of the adolescent intestinal microbiota under different living habits. All these factors influenced the intestinal microbiota at the genus level. Regarding dietary preference, adolescents who preferred to eat meat and had a balanced diet had more *Bacteroides* in the intestinal tract than those who preferred to eat vegetables (*p* < 0.05, FDR = 0.633; Figure 6A). With respect to flavor preference, *Escherichia*–*Shigella* were significantly more abundant in the intestinal tract of adolescents who preferred heavy seasoning than in that of those with a preference for light or balanced flavors (*p* < 0.05, FDR = 0.519; Figure 6B). *Dialister* is more common in the intestines of adolescents who sleep more (*p* < 0.05, FDR = 0.625; Figure 6C). However, exercise duration had an influence on the intestinal microbiota of adolescents. The abundance of *Faecalibacterium* increased with exercise duration (*p* < 0.05, FDR = 0.765; Figure 6D). However, these differences disappeared after FDR correction.

**Figure 7:**
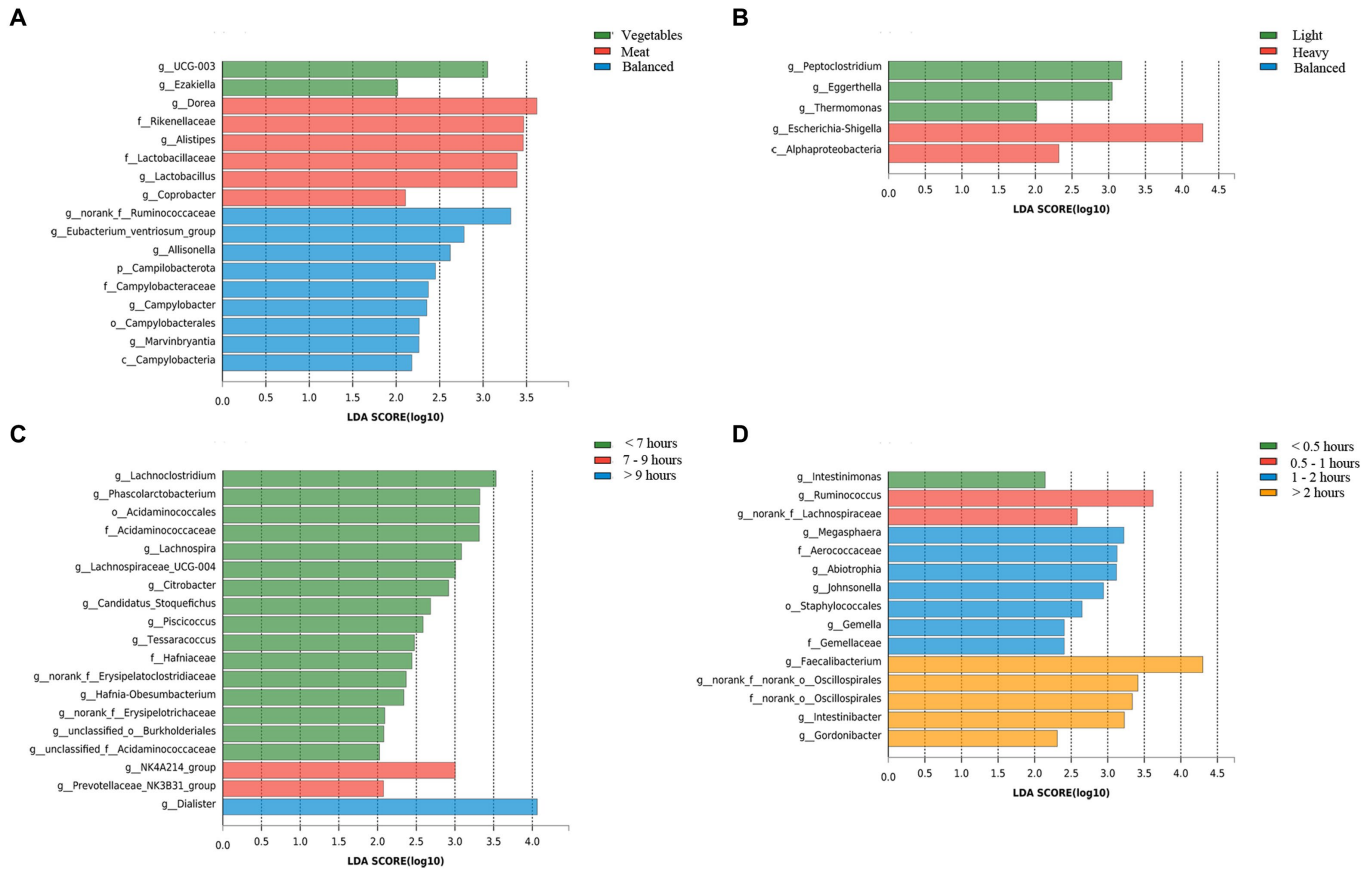
LefSe analysis of the intestinal microbiota from adolescents with different lifestyle habits. **(A)** Dietary preference; **(B)** Flavor preference; **(C)** Sleep duration; **(D)** Exercise duration.

In addition, LEfSe analysis was used to identify the key intestinal bacteria contributing to community structure differences in adolescents with different lifestyle conditions. The intestinal microbiota of adolescents with a light flavor preference, and strong flavor preference was characterized by the *Peptoclostridium* (LDA = 3.180, *p* = 0.003), and *Escherichia-Shigella* (LDA = 4.285, *p* = 0.02), respectively. Adolescents with different dietary preferences also had different intestinal microbiota community structures. *Ruminococcaceae* (LDA = 3.321, *p =* 0.03) was the specific genus in the intestines of adolescents with a balanced diet. In adolescents who preferred meat, the specific bacterium was *Dorea* (LDA = 3.622, *p* = 0.04), and *UCG-003* (LDA = 3.055, *p* = 0.02) was the specific strain in those who preferred to eat vegetables. The intestinal microbiota of adolescents who slept for less than 7 h, 7–9 h, more than 9 h was characterized by the *Lachnoclostridium* (LDA = 3.523, *p* = 0.002), *NK4A214_group* (LDA = 3.006, *p* = 0.012), and *Dialister* (LDA = 4.066*, p =* 0.03), respectively. In the analysis of exercise durations, *Intestinimonas* (LDA = 2.146, *p* < 0.001), *Ruminococcus* (LDA = 3.622623, *p* = 0.001), *Megasphaera* (LDA = 3.223, *p* = 0.01), *Faecalibacterium* (LDA = 4.303, *p* = 0.04) are the specific bacterium in the intestines of adolescents who exercise for less than half an hour, half an hour to 1 h, 1–2 h and more than 2 h, respectively (Figure 7).

## 4. Discussion

Previous studies have found regional differences in the human intestinal microbiota. This high abundance of intestinal *Bifidobacterium* has also been observed in Japanese children and is believed to be linked to the Japanese diet (Nakayama et al., 2015). People from western countries consume more fat and protein. Studies have found that these individuals show a higher abundance of *Bacteroides* in their intestinal microbiota. Meanwhile, these individuals typically consume carbohydrates in their simplest forms *via* drinks, cakes, desserts, and white bread. Hence, western diets are often high in energy and low in nutritional value, altering the metabolism of intestinal microorganisms and increasing the risk of obesity, cardiovascular diseases, and diabetes (Yatsunenko et al., 2012; Sampson and Mazmanian, 2015). In contrast, the long-term consumption of complex carbohydrates (such as dietary fiber) has been proven to increase the abundance of *Prevotella*. Therefore, the amount of intestinal *Prevotella* is low among westerners, while being higher among populations that consume coarse grains, such as people from Malawi, American Indians, and people from African countries (De Filippo et al., 2010; Ayeni et al., 2018). The intestinal microbiota of people from different areas shows vast differences. In our study, it also shows consistency. The diversity of intestinal microbiota of adolescents in urban areas is higher than that in town and rural areas, and the composition of intestinal microbiota of adolescents in urban is different from that in town and rural area. Living in different geographical areas may strongly affect the diversity of the intestinal microbiota during childhood.

The ratio of *Firmicutes* to *Bacteroidetes* is thought to be related to intestinal inflammation and obesity (Stojanov et al., 2020). Higher F/B ratios are linked to a higher risk of obesity (Ruth et al., 2005; Kasai et al., 2015; Crovesy et al., 2020) and lower risk of IBD (Manichanh et al., 2006; Walker et al., 2011; Kabeerdoss et al., 2015). Previous studies have found that the F/B ratio is higher among children living in urban Europe than in those from rural Africa (De Filippo et al., 2010). Researchers believe that this is due to the high protein and fat consumption among European children. However, the influence of the F/B ratio on diseases is still controversial. In many studies, this ratio has shown no correlation with obesity, and even the opposite conclusion has been reached (Castaner et al., 2018; Vaiserman et al., 2020). Our data showed that the abundance of *Actinobacteria* was higher than that of *Bacteroidota* in adolescents from rural regions and towns. *Bacteroidota* was significantly more abundant in adolescents from urban regions than in those from other regions. The F/B ratio was found to differ among adolescents living in cities, towns, and rural areas. Unexpectedly, it was lower in adolescents living in urban areas than in those from rural areas. However, as mentioned above, the dietary habits in the urban areas of Hangzhou were not similar to those observed in western cities. While meat consumption was prevalent in the city of Hangzhou, the high-frequency intake of vegetables and fruits was also observed. Hence, the relationship between the intestinal microbiota in adolescents from different regions of Hangzhou and obesity needs further analysis. At the genus level, many bacteria showed differences among adolescents from different types of areas. Among them, *Bifidobacterium* showed a lower abundance in adolescents from urban areas, *Bacteroides* showed a higher abundance, consistent with the intestinal microbiota observed in westerners (De Filippo et al., 2010). Hence, urban development had some influence on the intestinal microbiota of adolescents. The relative abundance of *Bifidobacterium* and *Bacteroides* was found to be higher in the intestinal tracts of children living in Bangkok, while that of *Prevotella* was higher in the intestinal tracts of children living in Khon Kaen. This was attributed to the lower frequency of vegetables and fruits consumed by children in Bangkok (Nakayama et al., 2015).Our data showed that the relative abundance of *Prevotella* is low in all three regions, but it is higher in rural areas. *Prevotella* abundance is linked to a low-fat and high-fiber diet. Traditional eating habits are maintained more frequently in rural areas, which have a relatively backward economy. Our analysis of dietary habits showed that adolescents in rural areas of Hangzhou preferred to eat more vegetables, resulting in the characteristic higher abundance of *Prevotella* and lower abundance of *Bacteroides.* In economically developed urban areas, the high protein and fat intake led to a significant increase in *Bacteroides*. The difference of intestinal microbiota among adolescents living in different areas may be related to their lifestyle habits including dietary.

Diet has long been considered an important determinant of the intestinal microbiota. Our study also found that there is a close connection between dietary habits and the intestinal microbiota in adolescents. There are more *Dorea* in the intestines of adolescents who prefer to eat meat than those in the other two groups. In previous studies, it was found that the abundance of *Dorea* in Type 2 Diabetes individuals increased significantly, and it was negatively correlated with the abundance of butyrate-producing bacteria (Li et al., 2020). The increase of *Dorea* may play a role in the development of Type 2 Diabetes. This shows that the preference for eating meat in adolescence can affect the metabolism of the body by changing the intestinal microecological environment. In other words, a balanced diet is conducive to the formation of a healthy microecological intestinal environment. Moreover, 71.6% of adolescents in urban areas had a balanced diet, which might explain the higher diversity of the intestinal microbiota in this population. So far, there has been limited research on the relationship between flavor preference and the intestinal microbiota among adolescents. Our research provides new insights into this area. Through CCA analysis, Flavor preference is the lifestyle habits that have the greatest influence on the intestinal microbiota of adolescents in Hangzhou. We also found that adolescents who consumed more seasoning-rich food had the highest abundance of *Proteobacteria*, especially *Escherichia*–*Shigella*. However, some probiotics were enriched in adolescents who consumed more balanced and light flavors. Previous studies have considered that prevalence of the bacterial phylum *Proteobacteria* is a marker for an unstable microbial community (dysbiosis) and a potential diagnostic criterion for disease (Shin et al., 2015). Intestinal microbiota disorders also usually involve *Escherichia*–*Shigella*, which are linked to various diseases, such as diarrhea, Type 2 Diabetes, and acute pancreatitis (Thingholm et al., 2019; Zhu et al., 2019; Mizutani et al., 2021). This suggests that lightly flavored food can help adolescents develop a healthy intestinal microbiota and prevent diseases.

We found that sleep duration is also related to the intestinal microbiota. The intestinal microbiota was different in adolescents who slept for less than 7 h. *Bifidobacterium* was more abundant in the group with a lower sleep duration, while *Bacteroides* was more abundant in adolescents who slept for longer, although the difference was not significant, while the abundance of *Dialister* is significantly increased in the adolescents with longer sleep time. In our study, we also found that adolescents in urban areas slept longer than those in rural areas, and the abundance of *Dialister* of adolescents in urban areas is higher. This shows that the difference of sleep duration may also have an impact on the intestinal microbiota of adolescents in different areas. Previous studies have found that vegetarianism is a risk factor for mental disorders (Johannes Michalak and Jacobi, 2012). This may be due to the low tissue concentrations of long-chain *n*–3 fatty acids and vitamin B12 in vegetarians, which may increase the risk of major depression (Kwok et al., 2002; Wolfgang Herrmann, 2002). Patients with mental disorders often have a variety of sleep problems, such as sleep interruptions and a reduced duration of sleep (Baglioni et al., 2016). Both *Dialister* related to carbohydrate metabolism (Gonçalves et al., 2016) and *Bacteroides* related to fat metabolism (Li et al., 2022) were increased in the intestinal tract of adolescents who slept longer, which might be the reason for the different distribution of intestinal microbiota in adolescents with different sleep duration. Given that adolescents are in a critical period of growth and development, a balanced diet could not only help in maintaining a stable intestinal microbiota but also promote a healthy psychological state and improve sleep quality.

Previous studies have found that exercise plays an important anti-inflammatory role in the body, and regular exercise also affects the distribution of intestinal flora. People with exercise habits had a greater diversity of gut microbiota than people without (O’Sullivan et al., 2015). This may be because exercise reduces the effects of stress-induced intestinal barrier dysfunction. Moderate exercise is also associated with a lower degree of intestinal permeability, preservation of mucus thickness, and a lower rate of bacterial translocation (Luo et al., 2014). Our study also found a correlation between exercise and the intestinal microbiota of adolescents. Individuals who exercised for a long time had more *Faecalibacterium*. In previous studies, probiotics such as *Bifidobacterium* and *Faecalibacterium* have been found to be negatively correlated with body weight (Remely et al., 2016; Barratt et al., 2022). In addition, low *Faecalibacterium* abundance has been detected in some patients with chronic diseases (Sedighi et al., 2017; Dominika Salamon et al., 2018; Gao et al., 2018). One explanation is that the microbial microbiota promotes the biotransformation of bile acids to regulate fat digestion and metabolism, decreasing serum lipid levels (Ji et al., 2012). In our study, *Faecalibacterium* was more abundant in individuals who exercised for a long duration. This indicated that a longer exercise duration may accelerate metabolism in adolescents by improving their intestinal microbiota. Adolescents who exercised for a long duration were more common in rural areas, and individuals from rural areas also had a higher abundance of probiotics. Hence, exercise durations could have also had a significant impact on the distribution of the intestinal microbiota in adolescents from different regions.

Our findings provide new ideas for exploring the characteristics of the intestinal microbiota in adolescents living in areas with different urbanization levels and the factors that influence these characteristics. Nevertheless, our study has some limitations. First, this study explored dietary habits and sleep durations using a survey questionnaire. Hence, the results may be subject to some conformity and recall bias. Second, we did not measure some specific blood gut microbiota-related metabolites, which may help us to further explain the existing research results. Third, the differences provided in this study are nominal significance, so they should be carefully explained. Fourth, as 16S rRNA amplicon sequencing does not allow species-level resolution, more detailed analysis is needed to explore the relationship between urbanization and intestinal microbiota in the future.

## 5. Conclusion

A total of 302 adolescents participated in this study, which was the first comprehensive study examining the influence of urbanization and living habits on the intestinal microbiota of Chinese adolescents. The findings suggest that the diversity of intestinal microbiota is higher among adolescents from urban areas than in those from towns and rural areas. These differences are related to dietary preferences, flavor preferences, sleep duration, and exercise duration. Our results provide a scientific basis for the development of strategies to enable the maintenance of a healthy intestinal microbiota in adolescents living in areas with different levels of urbanization.

## Data availability statement

The datasets presented in this study can be found in online repositories. The names of the repository/repositories and accession number(s) can be found at: https://www.ncbi.nlm.nih.gov/, PRJNA856408.

## Ethics statement

The studies involving human participants were reviewed and approved by the Ethics Committee of Hangzhou Center for Disease Control and Prevention (No. 20047). Written informed consent to participate in this study was provided by the participants’ legal guardian/next of kin.

## Funding

This work was supported by Hangzhou Agricultural and Society Development Project (Grant no. 202004A20).

## Author contributions

GZ: funding acquisition, data curation, formal analysis, and writing—original draft. LX: data curation, formal analysis, and methodology. YW and BW: data curation and formal analysis. WT, ZS, QK, and WL: sample collection. XP: methodology, supervision, project administration, and review. HM: methodology, supervision, project administration, and review. All authors contributed to the article and approved the submitted version.

## Conflict of interest

The authors declare that the research was conducted in the absence of any commercial or financial relationships that could be construed as a potential conflict of interest.

## Publisher’s note

All claims expressed in this article are solely those of the authors and do not necessarily represent those of their affiliated organizations, or those of the publisher, the editors and the reviewers. Any product that may be evaluated in this article, or claim that may be made by its manufacturer, is not guaranteed or endorsed by the publisher.

## Footnotes

^1^http://huttenhower.sph.harvard.edu/galaxy/root?Tool_id=lefse_upload

^2^www.majorbio.com
