# Supplementary figures and images for: Effects of urbanization and lifestyle habits on the intestinal microbiota of adolescents in eastern China
Source: Front Microbiol. 2023 Jun 12;14:989303. doi: 10.3389/fmicb.2023.989303 (PMC10291051; doi:10.3389/fmicb.2023.989303)

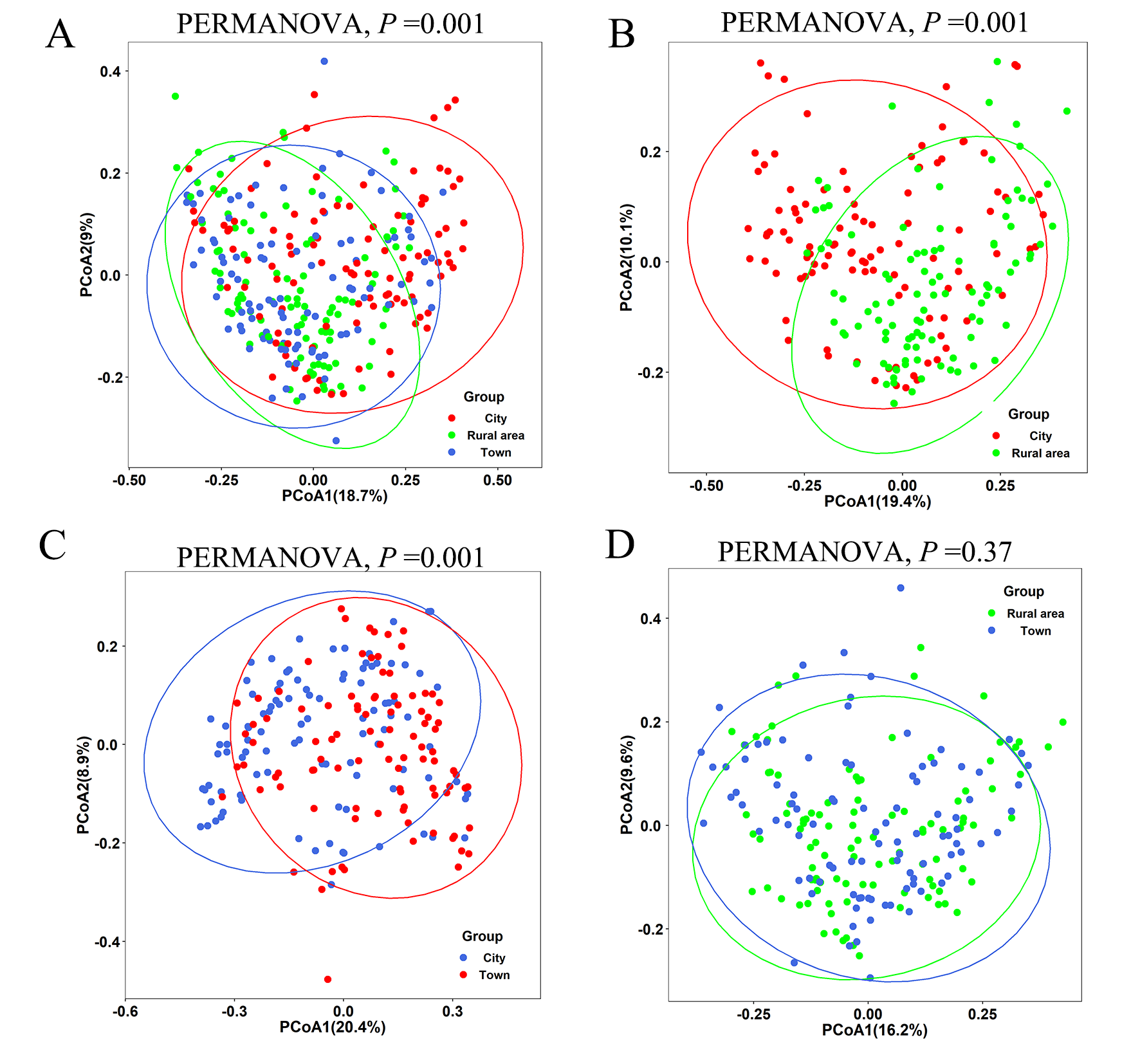

Supplement: Supplementary file 1 [file Image_1.TIF]

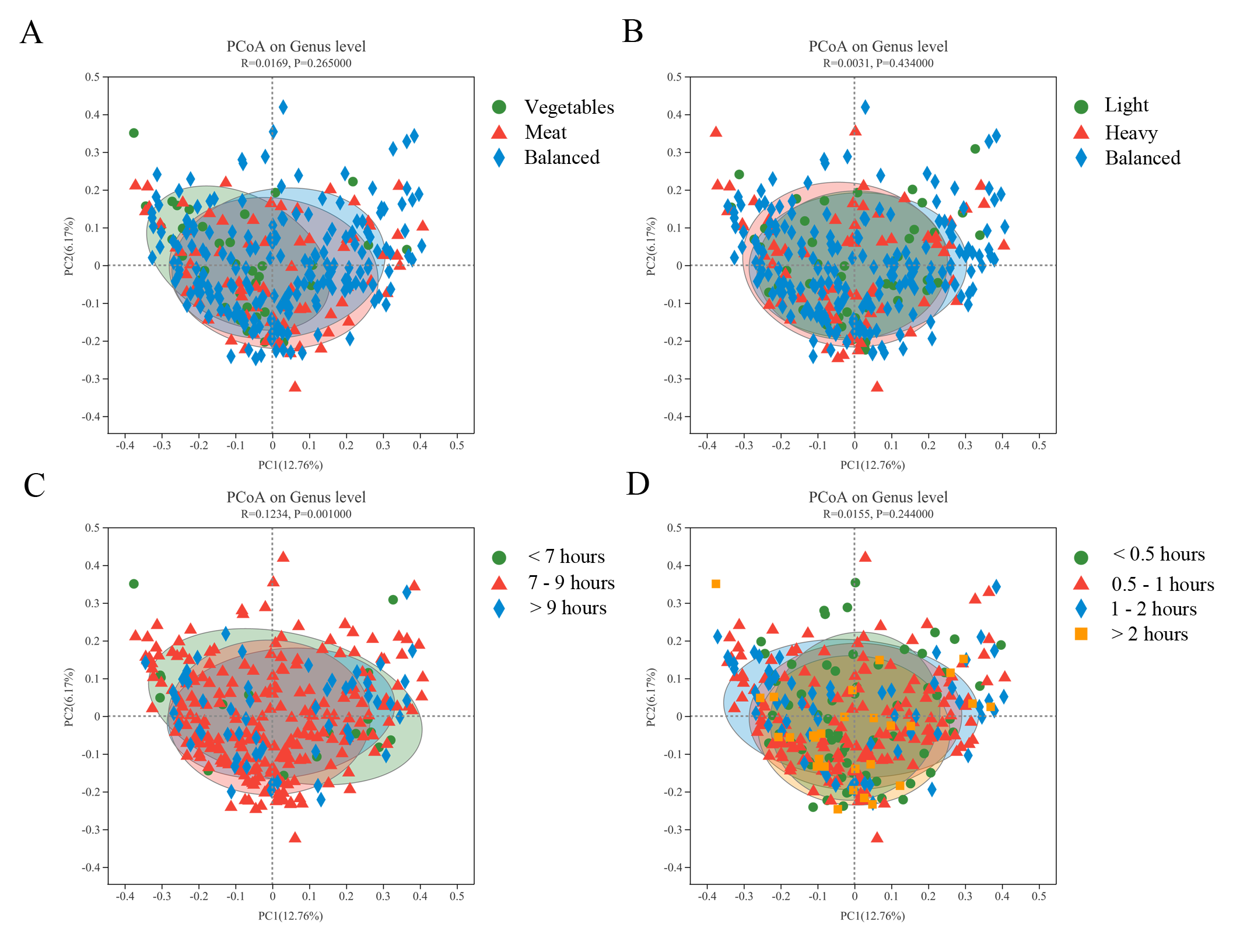

Supplement: Supplementary file 2 [file Image_2.TIF]
